# High-energy traumatic spondyloptosis at T8–T9 with complete spinal cord injury: a case report

**DOI:** 10.3389/fsurg.2025.1704439

**Published:** 2026-02-26

**Authors:** Ai-Jun Song, Chang-Feng Fu, Yuan-Yi Wang, Ya-Dong Liu, Jin-Wei Qi, Yan-Dong Li, Ying Zhao, Xu Feng

**Affiliations:** 1 Department of Spine Surgery, Orthopedic Center, The First Hospital of Jilin University, Changchun, China; 2 Jilin Engineering Research Center of Spine and Spinal Cord Injury, Changchun, China

**Keywords:** high-energy trauma, precision therapy, spondyloptosis, thoracolumbar fracture, three-column injury

## Abstract

**Introduction:**

Spondyloptosis, the most severe form of spondylolisthesis, involves complete (>100%) anterior or posterior displacement of one vertebra over the subjacent segment, resulting in total anatomical dislocation. Typically caused by high-energy trauma, it leads to severe spinal instability, bony fragment intrusion into the canal, and significant neurological deficits. This report presents a representative case of T8-T9 spondyloptosis with complete spinal cord injury [American Spinal Injury Association (ASIA) Impairment Scale Grade A]to analyze its injury features, surgical approach, and clinical outcomes.

**Patient concerns:**

A 61-year-old female was admitted to the hospital presenting with severe thoracodorsal pain and complete paralysis of both lower extremities for 8 h following a crushing injury by a heavy object. The patient exhibited intense back pain and a pronounced thoracic kyphotic deformity. Complete loss of motor and sensory function was observed below the xiphoid process level. Imaging studies revealed complete dissociation between the T8 and T9 vertebral bodies. The distal fracture segment (T9) was displaced posteriorly and superiorly, resulting in impaction of the anterior margin of the T9 vertebral body against the spinous process of T8. Complete fractures with rotational displacement were noted in the posterior elements, including the pedicles and facet joints at the T8–T9 level.

**Primary diagnosis:**

T8–T9 spondyloptosis with complete spinal cord injury (ASIA A).

**Interventions:**

On the ninth day post-injury, the patient underwent posterior open reduction, laminectomy for decompression, inter-laminar bone grafting, and segmental instrumentation with internal fixation of the thoracic fracture.

**Outcomes:**

The patient's postoperative vital signs remained stable. Imaging revealed satisfactory correction of the thoracolumbar deformity, adequate positioning of the internal fixation hardware, near-complete restoration of the spinal physiological curvature, satisfactory fracture reduction, reconstitution of the spinal canal morphology, and appropriate alignment of the implants, all of which met preoperative expectations.

**Conclusion:**

This case represents the first reported instance of T8–T9 spondyloptosis with complete spinal cord injury resulting from high-energy trauma. The management of high-energy thoracolumbar fractures necessitates an in-depth understanding of the injury mechanism to formulate an individualized surgical strategy.

## Introduction

Spondyloptosis is defined as a complete, greater than 100% anterior or posterior displacement of one vertebral body relative to the adjacent caudal vertebra. This condition represents the most severe form of spondylolisthesis, resulting in complete dislocation of the superior vertebra from its normal anatomical position. High-energy trauma-induced spondyloptosis is among the most challenging injury patterns in spinal surgery, typically caused by high-kinetic-energy mechanisms such as motor vehicle accidents, falls from height, or crush injuries (1, 2). This injury leads to a disruption of all three spinal columns, accompanied by varying degrees of vertebral comminution, dislocation, and spinal canal compromise. It frequently causes spinal cord or nerve root injuries, resulting in high rates of disability and necessitating complex clinical management (3, 4). Advances in imaging modalities and internal fixation devices have evolved surgical goals beyond mere neural decompression and mechanical stabilization. Contemporary objectives now emphasize precise anatomical reduction, reliable biomechanical reconstruction, and the maximization of neurological function recovery (5, 6). However, due to the highly heterogeneous nature of injury patterns and complex regional anatomy, achieving effective spinal canal decompression while simultaneously restoring spinal alignment and stability during reduction remains a significant challenge in clinical practice and an active area of research (7). This article analyzes a representative case of severe T8–T9 spondyloptosis to elucidate its injury characteristics, surgical strategy, and mid-term outcomes, aiming to provide insights for standardizing the diagnosis and treatment of this complex traumatic injury.

## Patient information

### Patient concerns

A 61-year-old female was admitted to the hospital presenting with severe thoracodorsal pain and complete paralysis of both lower extremities, which occurred immediately after being struck and presented to our hospital 8 h post-injury. The injury mechanism involved a direct impact to the mid-upper thoracic region from a falling freight elevator while the patient was walking in a supermarket aisle. This immediately resulted in severe, tearing-type thoracodorsal pain. A neurological assessment revealed complete loss of motor function below the xiphoid process level, accompanied by the absence of pain, temperature, light touch, and deep sensation, in addition to bowel and bladder incontinence. Physical examination revealed a pronounced kyphotic deformity at the T7–T9 vertebral levels. Clinical signs included widened interspinous spaces with a palpable step-off deformity, alongside positive percussion and tenderness upon palpation. Neurological examination demonstrated complete paraplegia, with muscle strength in both lower extremities graded at 0/5. Both patellar and Achilles tendon reflexes were absent. Loss of sensation in the perianal and perineal regions was observed, consistent with a diagnosis of complete spinal cord injury.

### Imaging findings

**Figure 1:**
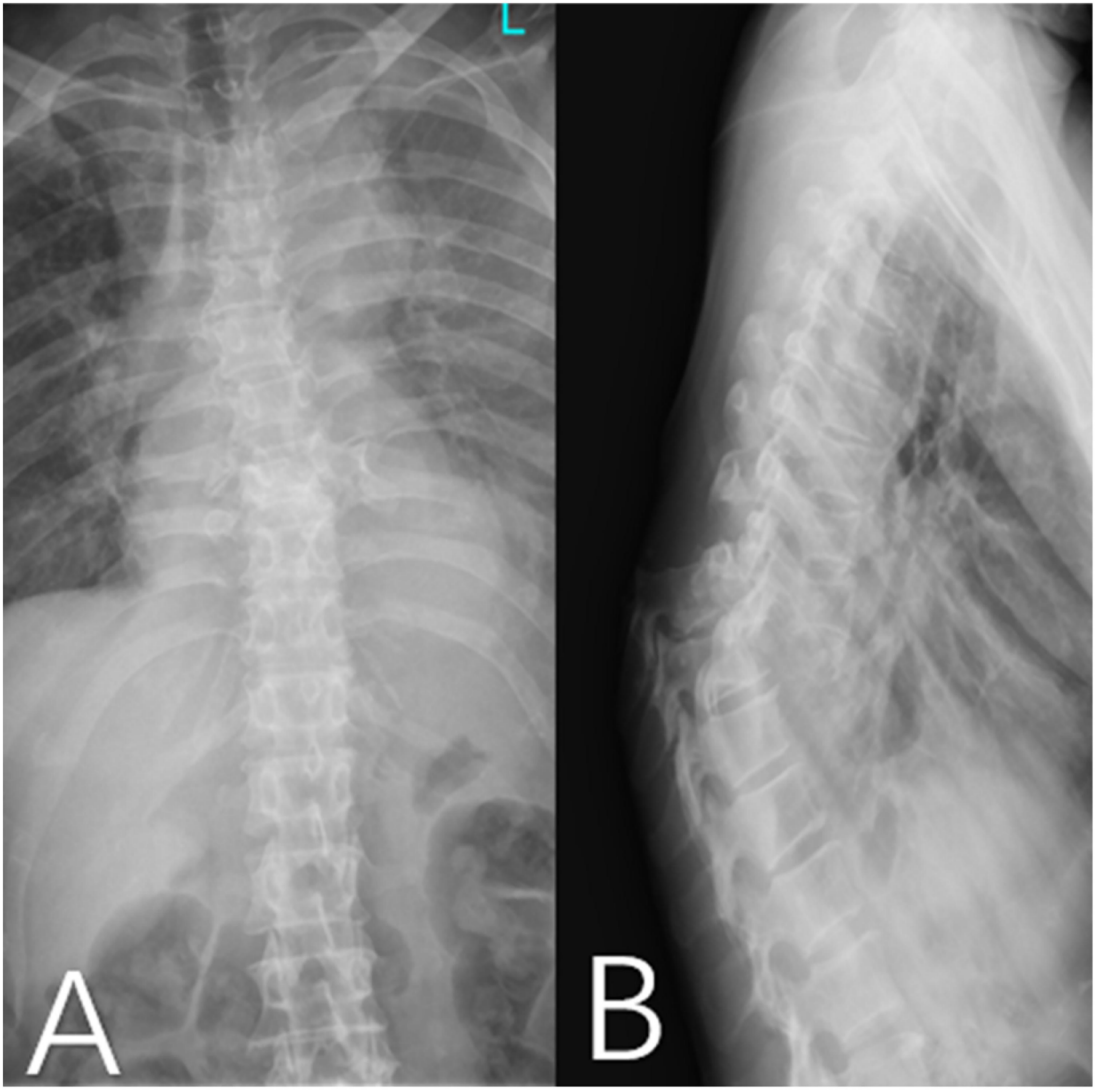
**(A)** Anteroposterior and **(B)** lateral radiographs of the thoracic spine demonstrate a fracture-dislocation at the T8-T9 segment. The distal fracture fragment is displaced posteriorly and superiorly, accompanied by a marked kyphotic deformity. Additionally, fractures of the T8 and T9 pedicles are evident, characterized by interruption and asymmetry of the pedicular contours, indicating severe disruption of the three-column spinal structure.

**Figure 2:**
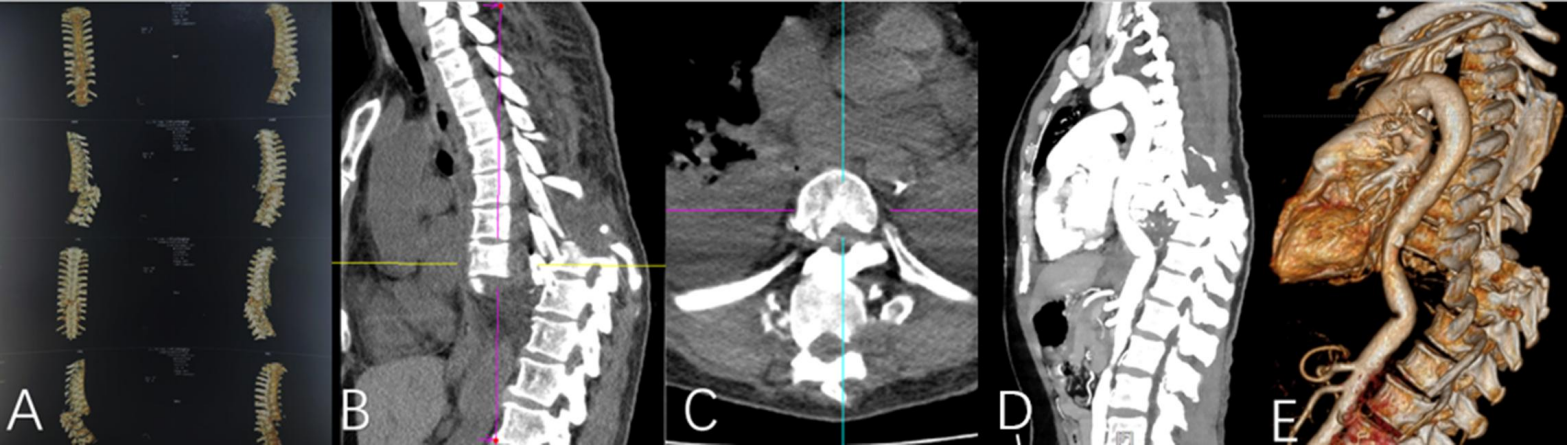
Thoracic spine **(A–E)** computed tomography (CT) images clearly demonstrate the severe spinal injury and adjacent vascular relationships at the T8–T9 level. A complete fracture-dislocation is observed between the T8 and T9 vertebral bodies. The distal fracture fragment is displaced posteriorly by approximately the combined width of one vertebral body and its posterior elements, accompanied by superior displacement of about one vertebral body height in the vertical plane. Complete fractures are evident in the posterior elements at the corresponding level, including the pedicles and facet joints. Additionally, free bone fragments are visible below the proximal fracture segment.

**Figure 3:**
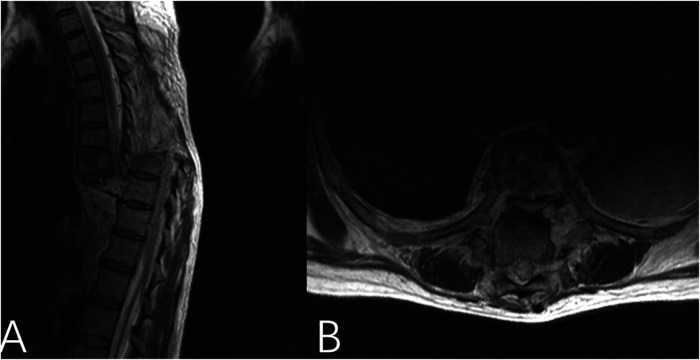
Magnetic resonance imaging (MRI) sequences **(A,B)** demonstrate complete discontinuity of the spinal column at the T8–T9 segment. Transection of the thecal sac and spinal cord is observed at this level, with interruption of the cerebrospinal fluid (CSF) signal. The proximal and distal spinal cord stumps have retracted, resulting in a conspicuous gap.

Imaging studies revealed a complete fracture-dislocation at the T8–T9 segment (Figure 1). The T9 vertebral body was displaced posteriorly and superiorly relative to T8 by approximately one vertebral body height, accompanied by a marked local kyphotic deformity. Computed tomography (CT) scans further confirmed complete dislocation between the T8 and T9 vertebral bodies. The T9 vertebral body was displaced posteriorly to the level of the proximal vertebral body, with the distal fracture segment displaced superiorly by approximately one vertebral body height in the vertical plane. Complete fractures were observed in the posterior elements, including the pedicles and facet joints. Free bone fragments were visible inferior to the proximal fracture mass, consistent with a three-column injury pattern resulting from high-energy trauma (Figure 2). Magnetic resonance imaging (MRI), T2-weighted sequences, demonstrated complete discontinuity of the spinal column at the affected segment. The thecal sac and spinal cord were completely transected, with interruption of cerebrospinal fluid signal. The proximal and distal spinal cord stumps had retracted, forming a distinct gap (Figure 3).

### Interventions

Upon admission, the patient presented with serious multiple trauma, massive hemorrhage, and unstable vital signs. These critical conditions rendered the patient unable to tolerate immediate surgical intervention, necessitating prioritized critical care support therapy to stabilize their physiological state. On the ninth day post-injury, the patient underwent open reduction of the thoracic fracture, laminectomy, and interlaminar bone grafting with internal fixation. During the procedure, the spinous processes, laminae, and facet joints from T6 to T10 were exposed, followed by the placement of bilateral pedicle screws at the T6–T10 levels. Following the implantation of the internal fixation, direct reduction was unsuccessful. Consequently, a partial inferior T8 vertebral corporectomy was performed to facilitate reduction via a pry-and-lever technique. Intraoperative visual inspection confirmed complete transection of the spinal cord. The bone cortex from T6 to T10 was decorrelated, and extensive bone grafting was performed for fusion of the facet joints across the same levels.

### Postoperative course

**Figure 4:**
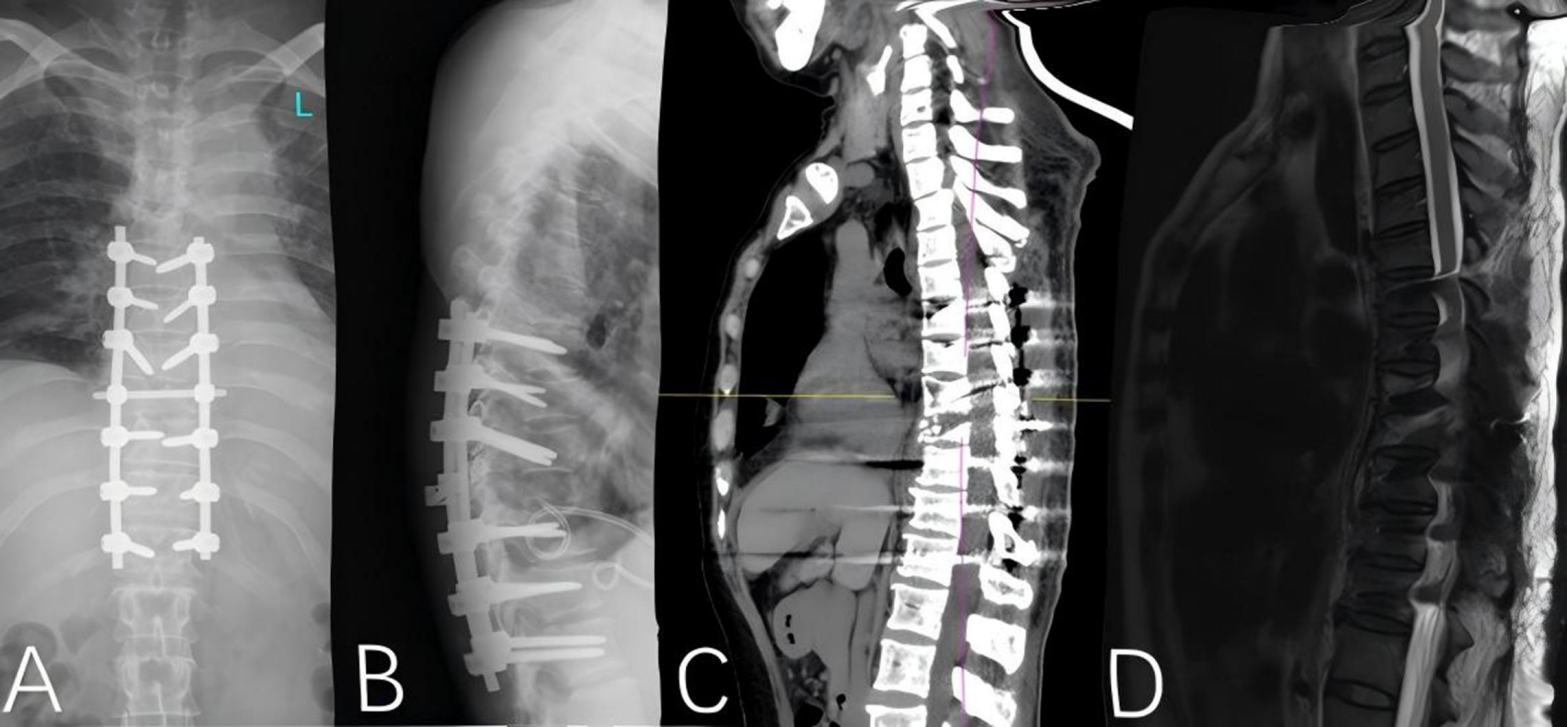
Postoperative digital radiographs of the spine, including **(A)** anteroposterior and **(B)** lateral views, demonstrate successful correction of the thoracolumbar deformity. The internal fixation hardware is well-positioned, and the physiological curvature of the spine is largely restored. Computed tomography (CT) and magnetic resonance imaging (MRI) scans **(C,D)** confirm satisfactory reduction of the fractured vertebral body.

The patient's postoperative condition was satisfactory, with stable vital signs. Imaging studies demonstrated successful correction of the thoracolumbar deformity, appropriate positioning of the internal fixation hardware, and substantial restoration of the physiological spinal curvature. Follow-up computed tomography (CT) and magnetic resonance imaging (MRI) revealed satisfactory fracture reduction, reconstitution of the spinal canal morphology, and alignment of the implants consistent with postoperative expectations (Figure 4).

## Discussion

Spondyloptosis, denoting complete vertebral dislocation, is classified as Grade V according to the Meyerding classification system (8). This represents the most severe form of spondylolisthesis, characterized by the superior vertebral body displacing more than 100% over the inferior one, resulting in its complete disengagement from the normal anatomical position. This condition carries a high risk of paralysis and other severe neurological deficits, necessitating surgical intervention for reduction and stabilization to restore spinal alignment and prevent further neurological compromise. High-energy trauma, predominantly from mechanisms such as motor vehicle accidents and falls from height, can cause one vertebral segment to become severely impacted or embedded into the axial space of the adjacent vertebra.Due to its unique anatomical and biomechanical properties, the thoracolumbar junction (T12–L2) is particularly susceptible to fracture-dislocation injuries. In contrast, spondylolisthesis occurs more frequently at the lumbosacral junction.Traumatic spondyloptosis at the mid-thoracic level (T8–T9) is exceptionally rare, as this region is inherently stable due to the rigid support provided by the thoracic rib cage (9).

The injury mechanism in this patient primarily resulted from high-energy vertical force applied to the thoracodorsal region. This force was predominantly converted into horizontal shear stress, leading to a characteristic and sequential failure of spinal structures. The process initiated with acute avulsion of the anterior longitudinal ligament, followed by fracture or dislocation of the facet joints. Subsequently, a horizontal tearing injury occurred through the vertebral body or intervertebral disc space. Ultimately, the proximal vertebral body slid anteriorly, resulting in irreversible displacement due to the persistent high-energy force (10, 11).

The reduction procedure presents three primary challenges: Firstly, mechanical impaction occurs when the proximal fracture fragment's spinous process becomes lodged against the anterior margin of the distal vertebral body, substantially increasing the mechanical resistance to reduction maneuvers. Secondly, the inherent stability of the thoracic cage creates a complex three-dimensional biomechanical environment. The fracture site is subjected to multidirectional stress forces during reduction, making it difficult to achieve anatomical alignment through a single-plane maneuver. Consequently, comprehensive adjustment of the three-dimensional mechanical axis is necessary (12). Thirdly, the proximity to major vasculature, such as the thoracic aorta anterior to the fracture zone, presents a significant risk. Aggressive reduction techniques can easily lead to vascular laceration or thrombus formation. Therefore, intraoperative precision in controlling reduction force is paramount. Prolonged dislocation may induce blood flow stasis within the vertebral artery, potentially leading to thrombus formation. Subsequent reduction maneuvers can restore blood flow and dislodge any formed thrombi, thereby potentially causing embolic ischemic events (11).

In the face of such complex and changeable fracture dislocation, we have formulated the following five stepped reduction schemes:

1. Closed Axial Traction under Anesthesia: This initial maneuver utilizes the muscle relaxation achieved under general anesthesia. Axial traction is applied to partially restore spinal height and alignment, establishing a foundation for subsequent reduction steps. This technique is applicable for some cases of flexion-distraction injuries or facet joint dislocations.
2. Intraoperative Reduction via Rod-Screw Distraction: This is a core reduction technique commonly used in posterior approaches. Pedicle screws are inserted into the vertebrae above and below the injured level and connected with a rod. Distraction forces applied through this rod-screw system restore vertebral height. The ligamentotaxis effect facilitates indirect reduction of bone fragments retropulsed into the spinal canal, thereby alleviating spinal cord compression.
3. Intraoperative Reduction by Exaggerating the Deformity: This alternative strategy is employed when direct reduction proves difficult. The existing kyphotic deformity or dislocation is intentionally increased to disengage the “locked” fracture fragments, followed by a reduction maneuver. This technique requires meticulous execution to avoid exacerbating neurological injury.
4. Removal of Obstructing Tissue and Leverage Reduction: This method is indicated when reduction is directly obstructed by bone fragments or disc tissue. Partial laminectomy, resection of the posterior vertebral margin, or removal of a protruding disc is performed to directly decompress the spinal cord and create space for leverage instruments to perform direct reduction of the fracture fragments.
5. In Situ Fixation: It is pursued when all reduction attempts fail or the risk of forced reduction is deemed prohibitively high. The goal shifts to achieving rigid fixation and fusion in the deformed position to maintain spinal stability, albeit with a lower probability of neurological function recovery. The patient underwent surgery on the ninth day post-injury, which consisted of a posterior thoracic approach for open reduction, laminectomy, and interlaminar bone grafting with internal fixation. Following surgical exposure from T6 to T10, bilateral pedicle screws were initially placed. However, attempted reduction via distraction through the rod-screw system failed following implant installation, suggesting the potential presence of bony obstruction or severe interlocking at the fracture site. Given the intraoperative failure of direct distraction reduction, we proceeded decisively with the predetermined fourth strategy: partial inferior T8 vertebral corpectomy followed by leverage reduction. This maneuver aimed to directly remove bony obstructions from the reduction path, achieve reduction of the displaced vertebra under direct visualization, and consequently maximize spinal cord decompression and restoration of spinal alignment. Intraoperative findings revealed complete spinal cord transection, which was consistent with the patient's preoperative clinical presentation of complete spinal cord injury. Although neurological recovery was not anticipated, successful reduction and stabilization were crucial for pain alleviation, enabling early rehabilitation training, and preventing secondary deformities.

## Conclusion

This case report describes a rare instance of complete spondyloptosis at the T8–T9 segment accompanied by complete spinal cord injury, resulting from high-energy trauma. The injury mechanism primarily involved the conversion of vertical force into horizontal shear stress, resulting in avulsion of the anterior longitudinal ligament, fracture or dislocation of the facet joints, and complete disruption of the intervertebral structures. This represents the most severe form of a three-column spinal injury. The patient presented with clinical features of a complete spinal cord injury, which was confirmed by imaging studies revealing complete transection of both the thecal sac and the spinal cord.

The surgical management consisted of a posterior approach for open reduction, spinal canal decompression, pedicle screw instrumentation, and interlaminar bone grafting and fusion. Intraoperatively, severe impaction and interlocking of the fracture fragments were observed, which prevented direct reduction. Consequently, a partial inferior T8 vertebrectomy was performed to remove the bony obstruction, enabling successful reduction using a leverage technique. Although neurological recovery was not achievable, the procedure successfully accomplished anatomical realignment of the spinal column, reconstruction of the spinal canal morphology, and restoration of spinal stability. This outcome established a crucial foundation for early patient rehabilitation and the prevention of secondary deformities.

This case highlights that for such severe thoracolumbar fracture-dislocations, an individualized and stepwise surgical strategy, initiated early and based on a thorough understanding of the injury mechanism, is essential. Precise reduction, adequate decompression, and stable reconstruction are critical components for improving prognosis and preventing complications. Furthermore, intraoperative vigilance against vascular injury is imperative. The surgical plan must be adjusted flexibly if reduction proves difficult, including the decisive removal of obstructive bony structures when necessary to achieve the reduction goal.

## Data Availability

The datasets presented in this article are not readily available because of ethical and privacy restrictions. Requests to access the datasets should be directed to the corresponding author/s.
